# Plasma proteome analysis in HTLV-1-associated myelopathy/tropical spastic paraparesis

**DOI:** 10.1186/1742-4690-8-81

**Published:** 2011-10-12

**Authors:** Paul DW Kirk, Aviva Witkover, Alan Courtney, Alexandra M Lewin, Robin Wait, Michael PH Stumpf, Sylvia Richardson, Graham P Taylor, Charles RM Bangham

**Affiliations:** 1 Centre for Bioinformatics, Division of Molecular Biosciences, Imperial College, London, SW7 2AZ, UK; 2 Department of Immunology, Wright-Fleming Institute, Imperial College, London, W2 1PG, UK; 3 Department of Clinical Biochemistry, Imperial College NHS Healthcare Trust, St Mary's Hospital, London W2 1PG, UK; 4 Department of Epidemiology and Biostatistics, Imperial College, London, W2 1PG, UK; 5 Kennedy Institute of Rheumatology, Imperial College London, 65 Aspenlea Road, London W6 8LH, UK; 6 Department of Genitourinary Medicine and Communicable Diseases, Wright-Fleming Institute, Imperial College, London, W2 1PG, UK; 7 Current Address: Warwick Systems Biology Centre, Coventry House, University of Warwick, Coventry CV4 7AL, UK

## Abstract

**Background:**

Human T lymphotropic virus Type 1 (HTLV-1) causes a chronic inflammatory disease of the central nervous system known as HTLV-1-associated myelopathy/tropical spastic paraparesis (HAM) which resembles chronic spinal forms of multiple sclerosis (MS). The pathogenesis of HAM remains uncertain. To aid in the differential diagnosis of HAM and to identify pathogenetic mechanisms, we analysed the plasma proteome in asymptomatic HTLV-1 carriers (ACs), patients with HAM, uninfected controls, and patients with MS. We used surface-enhanced laser desorption-ionization (SELDI) mass spectrometry to analyse the plasma proteome in 68 HTLV-1-infected individuals (in two non-overlapping sets, each comprising 17 patients with HAM and 17 ACs), 16 uninfected controls, and 11 patients with secondary progressive MS. Candidate biomarkers were identified by tandem Q-TOF mass spectrometry.

**Results:**

The concentrations of three plasma proteins - high [β2-microglobulin], high [Calgranulin B], and low [apolipoprotein A2] - were specifically associated with HAM, independently of proviral load. The plasma [β2-microglobulin] was positively correlated with disease severity.

**Conclusions:**

The results indicate that monocytes are activated by contact with activated endothelium in HAM. Using β2-microglobulin and Calgranulin B alone we derive a diagnostic algorithm that correctly classified the disease status (presence or absence of HAM) in 81% of HTLV-1-infected subjects in the cohort.

## Background

Human T lymphotropic virus Type 1 (HTLV-1) persists lifelong in the host and is associated with two distinct types of disease: a range of chronic inflammatory diseases, of which the most commonly recognized is HTLV-1-associated myelopathy/tropic spastic paraparesis (HAM/TSP, abbreviated hereafter as HAM), and an aggressive T cell malignancy known as adult T cell leukaemia/lymphoma (ATLL). The cumulative lifetime risk of HAM ranges between 0.1% and 3% of infected individuals; the cumulative lifetime risk of ATLL ranges from 1% to 5%.

The strongest correlate of the risk of HTLV-1-associated inflammatory diseases such as HAM is the proviral load (PVL), i.e. the percentage of peripheral blood mononuclear cells (PBMCs) that carry the provirus [1,2]. The PVL remains approximately constant within each infected individual, but differs among individuals by over 1000 times. However, the range of PVL overlaps extensively between patients with HAM and asymptomatic carriers: although a PVL > 1% PBMCs is strongly associated with HAM, 50% of asymptomatic carriers also have a PVL > 1%, which reduces the value of this measure in the clinical diagosis of HAM. Furthermore, other clinical manifestions of HTLV-1 are less well-defined, and it is difficult to identify in a given case whether HTLV-1 infection is co-incidental or causative. There is therefore an urgent need for additional tools to aid in the diagnosis of HTLV-1-associated disease both clinically and epidemiologically.

The mechanisms of pathogenesis of the HTLV-1-associated inflammatory diseases such as HAM remain uncertain. To date, most virological and immunological markers of HAM correlate with proviral load, but do not differ between patients with HAM and asymptomatic carriers at a given PVL. Few factors have been identified that differ systematically between asymptomatic carriers and patients with HAM at a given proviral load: the frequency of certain lymphocyte subsets (HTLV-1-specific CD4^+ ^T cells [3,4]; FoxP3^+ ^CD4^+ ^T cells [5]; natural killer (NK) cells [6] and NKT cells [7]); the level of expression of HTLV-1 genes in fresh PBMCs [5,8-10]; and the pattern of integration of the HTLV-1 provirus in the host cell genome [11]. However, none of these parameters is useful in the differential diagnosis of HAM from other causes of spastic paraparesis, and these parameters give only indirect suggestions as to the pathogenesis of the inflammatory conditions such as HAM.

The aim of the present study was to identify plasma proteins whose concentration is associated with HAM or correlated with proviral load, to help in the differential diagnosis of HAM and to provide further clues as to the mechanism of pathogenesis of the inflammatory disease. In a two-stage case-control study, we used surface-enhanced laser desorption ionization time-of-flight mass spectrometry (SELDI-TOF-MS; abbreviated here as SELDI) to identify plasma protein biomarkers that distinguished patients with HAM from both asymptomatic HTLV-1 carriers and patients with progressive multiple sclerosis, which closely resembles HAM clinically. Three biomarkers were identified by tandem mass spectrometry. We derive algorithms to estimate the utility of these biomarkers in the differential diagnosis of HAM, and discuss their possible significance in the pathogenesis of the disease.

## Results

### Univariate analysis revealed 4 biomarkers of HAM

Four successive pairwise comparisons were carried out: HAM vs. AC; AC vs. U; HAM vs. U; and (HAM and AC) vs. U. The results in Table 1 show the molecular weights of the peaks identified in each comparison for the original and verification data sets respectively. The *p*-values returned by the Biomarker Wizard software were converted into *q*-values, which estimate the false discovery rate [12] and account for multiple testing.

**Table 1:** Protein peaks whose intensity differed significantly (FDR level 0.05) between the two  respective subject groups.

Original data set							
**HAM vs. AC**		AC vs. U^(^*^)^		**HAM vs. U**		**(HAM & AC) vs. U**	

**MW (kDa)**	***q*-value**	**MW (kDa)**	***q*-value**	**MW (kDa)**	***q*-value**	**MW (kDa)**	***q*-value**

**11.7**	5.6E-04			**11.7**	1.0E-04	**11.7**	3.7E-02
**11.9**	9.4E-04			**11.9**	2.7E-04	11.9	4.4E-02
**13.3**	1.5E-03			13.3	3.9E-04	12.7	4.4E-02
**14.7**	1.9E-02			**14.7**	1.5E-03	**13.3**	4.4E-02
17.6	2.6E-02			**25.4**	4.5E-02	**14.7**	4.4E-02
Verification data set							

HAM vs. AC		AC vs. U		HAM vs. U		(HAM & AC) vs. U	

MW (kDa)	*q*-value	MW (kDa)	*q*-value	MW (kDa)	*q*-value	MW (kDa)	*q*-value

**14.7**	2.1E-02	13.8	6.2E-04	13.8	6.7E-04	13.8	3.4E-05
**13.3**	2.1E-02	6.9	5.6E-03	**11.7**	6.7E-04	6.9	1.3E-03
**11.7**	2.1E-02	9.7	1.5E-02	**14.7**	1.6E-03	14.0	7.2E-03
**11.9**	2.1E-02	14.0	3.6E-02	17.4	4.0E-03	**11.7**	9.9E-03
8.8	2.1E-02	8.6	3.6E-02	79.1	4.0E-03	8.6	9.9E-03
				17.6	4.0E-03	13.9	1.3E-02
				6.9	4.0E-03	17.4	1.3E-02
				39.7	4.0E-03	17.6	1.3E-02
				13.3	5.3E-03	12.8	1.7E-02
				14.0	7.0E-03	79.1	1.7E-02

17.4	3.3E-02						

Results are ordered by *q*-value; for brevity only the first ten results with q-value < 0.05 are shown (complete tables are provided in Additional file 1, S4). Peaks that reached significance (q < 0.05) in both original and verification sets are indicated in bold. ^(^*^) ^No peaks differed significantly between the AC and U groups in the original data set.

**Figure 1:**
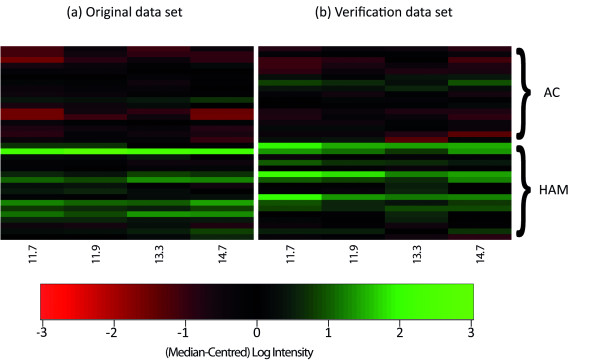
**Heatmap representation of intensities of the 11.7 kDa, 11.9 kDa, 13.3 kDa and 14.7 kDa peaks**. a) original data set; b) verification data set. Each row corresponds to a single subject; each column denotes a different protein peak. The colour depicts the log (peak intensity), after subtracting the median for each peak.

In the comparison of HAM vs. AC, four peaks remained statistically significant in both the original and verification data sets after multiple testing correction: the 11.7 kDa, 11.9 kDa, 13.3 kDa and 14.7 kDa peaks. As illustrated in Figure 1, the intensities of all four peaks were typically higher in the HAM group. The intensities of all four peaks in HAM patients also differed significantly from those in uninfected controls (Table 1; Additional File 1, S4).

### The plasma proteomic profile differed between HAM and multiple sclerosis

The samples from patients with HAM were compared with the samples from patients with secondary progressive MS. The results are shown in Table 2.

**Table 2:** Peaks that differed between HAM and MS samples, in descending order of statistical significance.

HAM vs. MS	
**MW (kDa)**	**q-value**

10.1	2.32E-04
3.8	2.32E-04
7.7	2.32E-04
9.2	2.32E-04
10.3	3.28E-04
37.4	3.28E-04
9.4	4.56E-04
10.8	4.78E-03
14.0	4.78E-03
60.4	4.78E-03

Only the first ten results are shown; complete data are provided in Additional file 1, S4.

Several peaks differed in intensity between the HAM and MS spectra (Table 2). The first four of these peaks (10.1 kDa, 3.8 kDa, 7.7 kDa and 9.2 kDa) were detected consistently only amongst the MS spectra; in the HAM spectra, their intensities fell below the noise level. For all but 2 of the peaks (9.4 kDa and 14.0 kDa), the mean intensity was higher in samples from patients with MS.

### Protein peak intensities were uncorrelated with proviral load

After correction for multiple testing, we found no peaks whose normalized log intensities remained significantly correlated with log(proviral load) in either the original or verification sets.

### SELDI data enabled HAM and AC to be distinguished with 79% cross-validation success rate

We aimed to identify protein peaks that discriminated between: (1) HAM and AC; and (2) HAM and MS.

1. **HAM vs. AC**. Three peaks (13.3 kDa, 11.7 kDa and 17.6 kDa) gave the lowest mean cross-validation (CV) misclassification rate (20.7%). The mean CV misclassification rate for the 11.7 kDa peak alone was 22.8%, and for 11.7 kDa and 13.3 kDa together was 21.4%.

2. **HAM vs. MS**. The 10.1 kDa peak alone gave the lowest mean CV misclassification rate of 6.6%, and further selections did not improve predictive performance.

**Figure 2:**
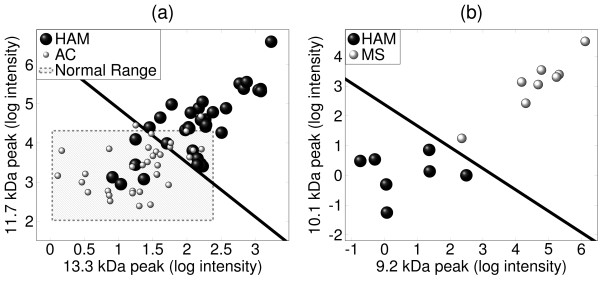
**Separation of (a) HAM and AC and (b) HAM and MS samples using the two most stably selected peaks**. In (a), the enclosed shaded region indicates a "normal range" of intensities for the two peaks, as determined from the uninfected controls.

The separation between the subject groups provided by the two most stably selected peaks in each of these cases is shown in Figure 2.

### Proviral load provided additional discriminatory information

Repeating the multivariate analysis of the HAM and AC classes including log(proviral load) as a predictor, we identified four parameters as significant discriminators: proviral load and the 13.3 kDa, 11.7 kDa and 11.9 kDa peaks. These four parameters combined gave a mean misclassification rate of 14.6%; the log(proviral load) alone provided a mean CV misclassification rate of 21.0%.

### A simple classification rule using the SELDI data

We constructed a classifier to discriminate between HAM and AC using only the 11.7 kDa and 13.3 kDa peaks, since these peaks were selected consistently in all analyses. For simplicity, we categorized the observed intensity values for each of these two peaks as either 'normal' or 'abnormal'. 'Normal' intensity measurements (Figure 2a, shaded region) were defined as those within 2 standard deviations of the mean amongst the uninfected controls (intensity = 3.2 ± 1.1 for the 11.7 kDa peak; 1.2 ± 1.2 for 13.3 kDa). From a logistic regression analysis of the resulting categorical data (see Additional file 1, S5), we derived the following rule:

"*If the intensity of either the 11.7 kDa or the 13.3 kDa peak (or both) is abnormal, then classify as HAM"*.

This rule correctly classified 55/68 = 81% of seropositive subjects in the 'combined' data set; this performance level may be overestimated, since the same data were used both to devise and assess the rule. Of the 13 misclassified individuals, 2 were ACs misclassified as HAM (i.e. false positive diagnosis of HAM), corresponding to a false positive rate of 5.9% and a false negative rate of 32.4%.

### Protein Identification

Because of their consistent significance in the statistical analysis, we wished to identify the proteins that constituted the 11.7 kDa and 13.3 kDa peaks. We also attempted identification of the 17.4 kDa and 14.7 kDa peaks (see Table 1).

**Figure 3:**
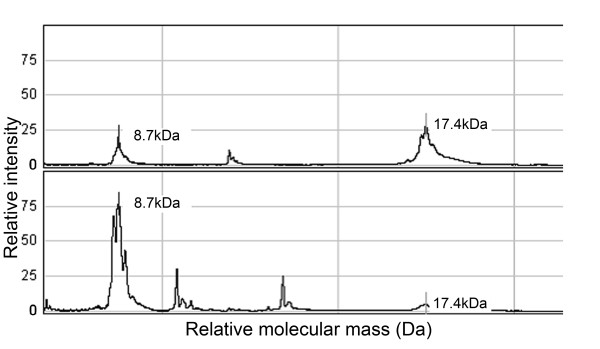
**Reduction of the putative ApoA-II homodimer (17.4 kDa) using DTT**. DTT was added to the passive eluate containing the 17.4 kDa protein and analysed on NP20 chips using SELDI-TOF-MS. (A) spectrum from eluate in absence of DTT; (B) spectrum from the same eluate after addition of DTT: the height of the 17.4 kDa peak was diminished and the peak at 8.7 kDa was correspondingly increased.

Q-TOF mass spectrometry identified the 17.4 kDa protein as apolipoprotein A-II (ApoA-II). The species present was presumably the S-S linked homodimer [13], whose calculated mass is 17.416 kDa, since the theoretical mass of its monomer (residues 24-100) is 8.708 kDa. This identification was confirmed by adding dithiothreitol (DTT) to the eluate containing the 17.4 kDa protein and repeating the SELDI analysis (Figure 3). Details of peptide fragments detected in the Q-TOF analysis are available on request.

Q-TOF analysis also identified the 11.7 kDa and 13.3 kDa protein eluates as β2-microglobulin (calculated mass 11.731 kDa) and Calgranulin B (Protein S100-A9) (calculated mass 13.224 kDa).

Purification of the 14.7 kDa protein by anionic fractionation and 1D-SDS-PAGE failed to resolve the peaks into separate gel bands. Three consistent peaks were detected by SELDI analysis. Q-TOF-MS analysis revealed three potential identities: transthyretin (calculated mass = 13761.4 Da); serum amyloid A-4 (SAA-4) (calculated mass = 12863.28 Da; glycosylation may increase the mass in vivo); and lysozyme.

### Confirmation of identity of β2-microglobulin

The plasma concentration of β2-microglobulin was measured in the subjects in the original sample set (17 patients with HAM, 17 ACs, 16 uninfected subjects) by rate nephelometry. The area under the 11.7 kDa peak was significantly correlated (Spearman rank correlation, 2-tailed) with the β2-microglobulin concentration in both the uninfected individuals (p = 0.023) and the asymptomatic carriers (p = 0.006); the correlation was suggestive in the patients with HAM (p = 0.09). The statistical significance of the correlation in the three subject groups combined was p = 0.0009, by Fisher's method of combining probabilities [14]. We conclude that the 11.7 kDa peak was formed by β2-microglobulin.

### Correlation between plasma β2-microglobulin concentration and disability in HAM patients

**Figure 4:**
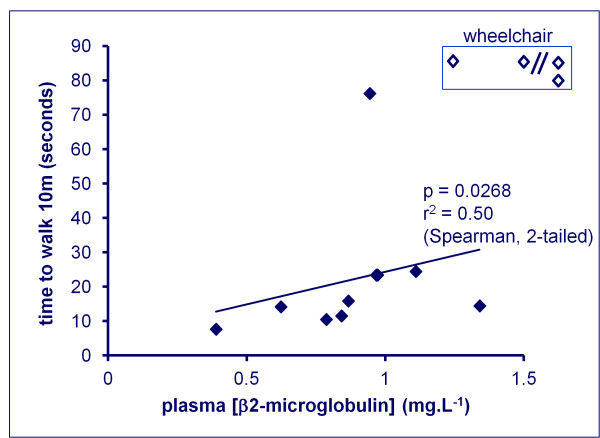
**In patients with HAM, the plasma concentration of β2-microglobulin (measured by rate nephelometry) was positively correlated with the time taken by the patient to walk 10 m**. Of the 5 patients with the highest plasma [β2-microglobulin], four were confined to a wheelchair (top right of figure, unfilled symbols; the two symbols to the right of the//mark represent values of 2.1 and 6.1 mg. L^-1 ^respectively).

A good objective measure of the disability caused by HAM is the time taken by the patient to walk 10 m on a flat, smooth surface. There was a significant positive correlation between the 10 m timed walk and the plasma β2-microglobulin concentration (Figure 4).

## Discussion

Biomarkers serve three chief purposes: to assist in the diagnosis of a clinical condition, to follow the progression of disease and response to treatment, and to give clues as to the pathogenetic mechanisms of the disease in question.

We identified two plasma proteins whose abundance in HTLV-1-infected individuals was significantly and independently associated with the presence of HAM: β2-microglobulin and Calgranulin B. Moreover, the plasma β2-microglobulin concentration was significantly correlated with the degree of disability resulting from HAM as measure by the 10 m timed walk (Figure 4). Two further peaks (11.9 kDa and 14.7 kDa) had intensities that were strongly correlated with the 11.7 kDa (β2-microglobulin) and 13.3 kDa (Calgranulin B) peaks respectively. However, inclusion of these peaks did not add significantly to the discrimination between asymptomatic HTLV-1 carriers and patients with HAM. A logistic regression model including the abundance of the two most strongly associated biomarkers, β2-microglobulin and Calgranulin B, correctly classified the clinical status of HTLV-1-infected subjects in the present cohort with 79% accuracy (i.e. CV misclassification rate of 21%). A 17.4 kDa protein peak, identified as Apolipoprotein-AII (Apo-AII), was also of particular interest as it was more abundant in asymptomatic carriers than in patients with HAM.

Two of the identified peaks (β2-microglobulin and Calgranulin B) discriminated between HAM and MS patients, with a CV misclassification rate of 25.0%. However, the best discrimination between patients with HAM and those with secondary progressive MS was provided by the protein peak at 10.1 kDa (Figure 2b): this protein awaits identification.

The serum concentration of β2-microglobulin is in clinical use as a marker of chronic renal disease, in risk stratification and as a measure of disease burden in certain haematological malignancies [15-17], and as a measure of disease activity and progression in HIV-1 [18] and HIV-2 infection [19]. In Alzheimer's disease, Simonsen *et al. *[20] found a lower concentration of β2-microglobulin in the cerebrospinal fluid (CSF) in patients compared with healthy age-matched controls, using SELDI techniques. In one study of multiple sclerosis, a higher concentration of β2-microglobulin was found in CSF in patients with active disease compared with healthy individuals [21]; however, this finding was not reproduced in subsequent studies, either in CSF [22,23] or in the serum β2-microglobulin concentration [23]. In patients with HAM, as in HIV-1 [18] and HIV-2 infections [19], it is likely that the high β2-microglobulin concentration in serum is a result of chronic activation of large numbers of T cells.

Calgranulin B [24,25] is also known as S100A9 and as myeloid-related protein 14 (MRP14). Together with the related protein Calgranulin A (S100A8; MRP8), Calgranulin B forms a heterodimer called Calprotectin, which plays a central part in calcium ion signalling during inflammation. Calgranulin B is expressed chiefly in neutrophils, and in monocytes after activation of protein kinase C. It is also expressed in some epithelial cells, but not in fixed tissue macrophages or in lymphocytes. Contact with activated endothelium induces monocytes to secrete Calgranulin B (by a microtubule-dependent mechanism), but contact with resting endothelium suppresses this secretion [26]. The concentration of Calgranulin B in serum (or other fluids such as synovial fluid) correlates with disease activity in a wide range of inflammatory conditions, including rheumatoid arthritis [27], pauciarticular onset juvenile rheumatoid arthritis [26]; inflammatory bowel disease [28]; chronic airway inflammation [29]; kidney allograft rejection [30] and acute appendicitis [31].

The strong association found in this study between serum Calgranulin B abundance and HAM suggests that monocytes are activated by contact with activated endothelium in patients with this disease. However, macrophages themselves may not contribute to the tissue damage seen in HAM: macrophages are not a prominent feature of the cellular infiltrate in CNS lesions [32].

Apolipoprotein-AII (Apo-AII) [33] is a major constituent of high-density lipoproteins (HDL). Castellani *et al. *[34] found that overexpression of Apo-AII in transgenic mice converted HDL to proinflammatory particles which induced transmigration of monocytes across the arterial wall, and these authors suggested a role of Apo-AII in atherosclerosis. Further evidence of an inflammatory role of Apo-AII was found by Thompson *et al. *[35], who showed that Apo-AII enhanced the monocyte response to bacterial lipopolysaccharide. The present results show that a high peak of Apo-AII was associated with asymptomatic carrier status. Further work is warranted to investigate the possible contribution of monocytes to the pathogenesis of HAM.

Semmes *et al. *[36] reported a SELDI analysis of serum proteins in HTLV-1 infection. These authors compared the SELDI proteomic profile between patients with ATLL, patients with HAM, and uninfected control subjects. They found that two serum proteins were overexpressed in ATLL: these proteins were identified as alpha-1-antitrypsin and haptoglobin-2 respectively by tandem mass spectrometry. The authors concluded that these two proteins could serve as biomarkers to distinguish between ATLL and HAM. However, these authors did not examine the serum proteome in asymptomatic HTLV-1 carriers or patients with multiple sclerosis, and the relationship between the protein peak intensity and the proviral load of HTLV-1 was not explored.

## Conclusions

The biomarkers identified here are potentially of clinical use, because they were specifically associated with the presence of the disease, HAM, and gave additional discriminatory information to that provided by the proviral load. These biomarkers may therefore serve both in the differential diagnosis of HAM and in following the disease activity and response to treatment in individual patients. The plasma (or serum) concentration of β2-microglobulin may be of particular clinical utility, because the assay is readily available in clinical chemistry laboratories and because the concentration correlates with clinical severity (degree of disability). The classification rule ("*if the intensity of either the 11.7 kDa or the 13.3 kDa peak - or both - is abnormal, then classify as HAM"*) provides a useful basis for further clinical testing. In addition, it will be interesting to investigate these biomarkers in other diseases that are known or suspected to be caused by HTLV-1, such as polymyositis, arthritis and uveitis.

## Methods

### Subjects; plasma samples

Plasma was prepared from EDTA-anticoagulated peripheral venous blood samples donated by a total of 95 subjects, comprising 68 HTLV-1 infected patients (34 patients with HTLV-1 associated myelopathy/tropical spastic paraparesis (HAM) and 34 asymptomatic HTLV-1 carriers (ACs)) and 16 ethnically matched uninfected controls (U) attending clinic at the National Centre for Human Retrovirology, St Mary's Hospital, London. These samples form part of the Research Tissue Bank (Imperial College London), approved by the UK National Research Ethics Service (09/H0606/106). EDTA-anticoagulated peripheral venous blood samples were also donated by 11 patients with secondary progressive multiple sclerosis attending Charing Cross Hospital, London. All patients gave written informed consent. Details of participating subjects (age, sex, ethnicity, disease status) are available on request.

### Sample processing

Samples were analysed by SELDI using the CM10 ProteinChip array (Bio-Rad, Hemel Hempstead, UK) with 50 mM sodium acetate buffer (pH4), and sinapinic acid as the energy-absorbing matrix. Data were collected at low- and high-mass ranges consecutively, using the manufacturer's protocol. The low-mass range was 1-30 kDa (highest mass collected 50 kDa) and the high-mass range 10-75 kDa (highest mass collected 100 kDa). For each subject we then combined the data in the optimal part of each mass range (1 to 10 kDa from the low-mass range and 10 to 100 kDa from the high-mass range). See Additional file 1, S1 for details.

### Processing of spectral data: Biomarker Wizard

Spectra were processed using the Ciphergen ProteinChip Software (version 3.2.0). Before statistical analysis with the proprietary software package Biomarker Wizard (Bio-Rad), spectra were calibrated using four molecular weight standards and normalized by intensity (total ion current). The spectra from each experiment were analysed as either low- or high-mass data, in two groups (HAM vs AC; AC vs U; HTLV-1^+ ^vs MS). Following the manufacturer's guidelines, criteria for protein peak detection were a signal to noise ratio (S/N) of ≥10 and presence of the peak in ≥40% of samples in at least one subject group. All spectra were inspected visually by an experienced SELDI operator; no spectra were omitted from the study.

### Experimental design

The study comprised three stages. In stage 1 we analysed samples from 17 patients with HAM, 17 ACs and 16 Us. In stage 2 we analysed samples from a non-overlapping cohort of 17 HAM patients and 17 ACs together with repeat aliquots of the plasma samples from 14 of the 16 Us studied in the first set. Finally, samples from 11 patients with MS were analysed together with 12 samples chosen randomly from the 34 patients with HAM. These three data sets were respectively denoted the 'original', 'verification' and 'MS' sets.

To derive predictive multivariate models, we pooled data from the 'original' and 'verification' sets to create a 'combined' data set comprising samples from all 68 HTLV-1-seropositive individuals, after further normalization to minimize bias due to chip-specific effects [37]. Further details are provided in Additional file 1, S2. As an alternative approach, we trained a predictive model using the 'original' dataset only, and then tested its performance on the 'verification' dataset. This method also yielded good results (see Additional file 1, S8). The workflow of sample processing and analysis is summarized in Additional file 1, S9.

### Univariate Analysis

#### Association with disease group

For each comparison of interest we identified significant differences (Mann-Whitney U test) in the distributions of peak intensities between the two respective subject groups.

#### Correlation with proviral load

We tested for a correlation (Kendall's τ rank correlation coefficient; exact *p*-values) between protein peak intensities and proviral load separated in samples from ACs and patients with HAM in the original and verification data sets.

#### Multiple testing correction

For all univariate tests, we controlled the false discovery rate at *q *= 0.05 [12,38].

### Multivariate Analysis

#### Prediction and peak selection

We used a logistic regression model to describe the probability of disease outcome. To fit the model to the data we used a maximum likelihood method incorporating the lasso penalty [39,40]. A stability selection approach [41] was used to select the peaks whose selection was robust to data variability. We selected the optimum number of peaks using a cross-validation assessment of predictive performance. Further details are provided in Additional file 1, S3.

#### Identification of candidate biomarker proteins

To identify the candidate proteins, plasma samples with a high abundance of the desired protein (i.e. high peak height on the SELDI spectrum) were selected. HAM samples were used to identify 11.7 kDa, 13.3 kDa and 14.7 kDa proteins, whereas AC plasma was used to identify the 17.4 kDa protein. Albumin-depleted plasma was subjected to successive anionic exchange fractionation and reverse phase fractionation; and separated by 1D-SDS-PAGE. Proteins at the target molecular weight were digested with trypsin (either in gel or after passive elution) and analysed using Q-TOF-MS/MS. All samples were run in duplicate. See Additional file 1, S6 for further details.

#### Protein identification by tandem mass spectrometry

Gel bands were excised and digested with trypsin as previously described [42]. Samples were analysed by high performance liquid chromatography coupled to electrospray ionization tandem mass spectrometry (HPLC ESI MS/MS using a Waters Q-TOF instrument). Proteins were identified by correlation of uninterpreted spectra to the SwissProt database (Release 2010_04) using Mascot (version 2.2: http://www.matrixscience.com). MS/MS ion searches specified up to two missed cleavages per peptide, a precursor mass tolerance of ± 100 ppm and a fragment ion mass tolerance of ± 0.5Da. Carbamidomethylation of cysteines and methionine oxidation were specified as fixed and variable modifications respectively.

Q-TOF MS/MS based peptide and protein identifications were validated using the Protein and Peptide Prophet algorithms, as implemeted in the program Scaffold version 3.01 [43] (Proteome Software Inc., Portland, Oregon). Peptide identifications were accepted if they could be established at greater than 95.0% probability. Protein identifications were accepted if established at greater than 99.0% probability and contained at least 2 matched peptides.

## Competing interests

The authors declare that they have no competing interests.

## Authors' contributions

PK contributed to the study design, carried out the mathematical, statistical and computational analysis and wrote the paper; AW carried out the sample preparation and SELDI analysis, and the data analysis using BioMarker Wizard; AC carried out the experimental work to identify candidate biomarkers; AL contributed to mathematical and statistical analysis; RW identified the candidate biomarkers by tandem mass spectrometry; MS and SR contributed to mathematical and statistical analysis; GP contributed to study design, clinical diagnosis, and writing the paper; CB conceived the study and contributed to study design, analysis and writing the paper. All authors read and approved the final manuscript.

## Supplementary Material

Additional file 1 **Study design, methods and results**. Additional file 1 contains further details on the following: study design; sample processing; chip-specific normalization; prediction and peak selection; complete significance tables; a simple classification rule using the SELDI data; differences in detection; protein identification (materials & methods); prediction using 'original' dataset as training set and 'verification' as test set; workflow summary; and references. Click here for file
