# Supplementary material for: Plasma proteome analysis in HTLV-1-associated myelopathy/tropical spastic paraparesis
Source: Retrovirology. 2011 Oct 12;8:81. doi: 10.1186/1742-4690-8-81 (PMC3210102; doi:10.1186/1742-4690-8-81)
Supplement: Additional file 1 — Study design, methods and results. Additional file 1 contains further details on the following: study design; sample processing; chip-specific normalization; prediction and peak selection; complete significance tables; a simple classification rule using the SELDI data; differences in detection; protein identification (materials & methods); prediction using 'original' dataset as training set and 'verification' as test set; workflow summary; and references. [file 1742-4690-8-81-S1.DOCX]

## Plasma proteome analysis in HTLV-1-associated myelopathy/tropical spastic paraparesis (P. Kirk et al.) Additional file 1

**Study design, methods and results**

### Study design

### Two-stage approaches such as ours have been used for some years [1], for example in the discovery of serum biomarkers of tuberculosis [2]. The procedure adopted here is distinct from the “protein pattern” method, in which spectral signatures are sought without recourse to protein identification [3]. In our protocol the SELDI data are only used to select candidate protein peaks; the relative imprecision with which SELDI assigns molecular masses to protein peaks does not impair this procedure [4]. Criticisms of SELDI have focused chiefly upon experimental protocols and statistical analyses [5]. We mitigate these issues by the following measures: (i) employing a randomized experimental design to preclude bias due to systematic variation between positions on the SELDI chip; (ii) checking conclusions using a verification data set; (iii) working with clustered protein peaks rather than raw spectral data; (iv) applying a range of statistical methods to verify reproducibility of results; and (v) ensuring that the protein peaks selected as candidates are stable (i.e. that the same candidates are selected even if the data are slightly perturbed).

### S1. Sample processing

**Sample preparation; SELDI conditions**

Samples were analysed by SELDI using ProteinChip arrays (Bio-Rad, Hemel Hempstead, UK). Preliminary experiments were carried out to determine the SELDI chip surface, buffer and energy-absorbing matrix (EAM) that gave the largest number of protein peaks on the spectrum. We selected the CM10 chip (weak cationic exchange surface) with 50mM sodium acetate buffer (pH4), with sinapinic acid as the EAM.

The ProteinChip array was washed twice with buffer before incubation with the samples at room temperature. The array was washed twice more with buffer and once with water prior to air-drying. A saturated EAM solution plus All-In-One Protein standard was added to the array before it was again air-dried and then placed in the ProteinChip Reader model PBSIIc (Ciphergen Biosystems, Freemont, CA, USA) for analysis.

Data were collected at low- and high-mass ranges consecutively, using the manufacturer’s protocol. The low-mass range was 1-30kDa (highest mass collected 50kDa) and the high-mass range 10-75kDa (highest mass collected 100kDa). For each subject we then combined the data in the optimal part of each mass range (1 to 10kDa from the low-mass range and 10 to 100kDa from the high-mass range). The laser intensity was set at 170 and 180 for the low- and high-mass runs respectively. Detector sensitivity was set at 8.

**Processing of spectral data: Biomarker Wizard**

The spectra were processed in separate experiment files using the Ciphergen ProteinChip Software (version 3.2.0), according to chip type, pH, EAM and mass range used. Before statistical analysis with the proprietary software package Biomarker Wizard (Bio-Rad), the spectra were calibrated using four molecular weight standards from the All-In-One Protein Standard II (Bio-Rad) and normalized by intensity (total ion current). Segments were set for measurement of local noise; the baseline was subtracted in all spectra and the baseline-fitting width was adjusted to 6 x expected peak width. The spectra from each experiment were analysed as either low- or high-mass data, in two groups (HAM vs AC; AC vs U; HTLV-1^+^ vs MS). Criteria for protein peak detection were a signal to noise ratio (S/N) of ≥10 and presence of the peak in ≥40% of samples in at least one subject group.

### S2. Chip-specific normalization

Chip effect parameters were estimated using only detected peaks from the control samples, which were run on every chip. We used a linear model of the type often used in analyses of microarray data [6]:

(1)

where is the measured log intensity of peak *i* in the spectrum from the control sample on chip *k*, is the true unobserved intensity of peak *i* in the control sample, is a chip-specific fixed effect and is a zero-mean random error term. The moment estimator of the chip effect is . We normalized intensities of all other spectra by subtracting the appropriate chip effect from the log intensities: where is the log intensity for peak *i* in sample *j*, and *k*(*j*) denotes the chip on which sample *j* was run.

### S3. Prediction and peak selection

We performed a multivariate analysis in order to identify the peaks whose intensities provide the greatest ability to discriminate between disease outcomes. We used a simple logistic regression model to describe the probabilities of class membership for each individual:

(2)

Here, the classes and denote the disease groups of interest (e.g. HAM and AC; or HAM and MS) and denotes the normalised log intensity of peak *i* for sample *j*. To fit our model to the observed data, we used a maximum likelihood method in which we incorporated the lasso penalty in order to shrink coefficients to zero and hence perform variable selection [7, 8]. That is, the vector of coefficients was estimated as:

(3)

where ℓ(β) denotes the log-likelihood function of the logistic regression model and λ is the shrinkage parameter. We considered the order in which the coefficients were selected as we reduced the level of shrinkage (by reducing λ), under the assumption that the variables which are selected first are the most important discriminators. We further employed a stability selection method (similar to that of Meinshausen and Bühlmann, [9]) to promote the inclusion of only those peaks whose selection is robust to data variability. We proceeded by repeatedly sampling 50% of the observed data at random in order to obtain 100 resampled data sets. For each of these, we fitted our lasso logistic regression model and noted the order in which variables were selected as we reduced the level of shrinkage. We then calculated the proportion of times (across all resampled data sets) that each variable appeared amongst the first *m* selections. For a given number *m* = *M* of selections, our final selections were those variables which were most frequently selected across all data sets (as these represent the selections which are maximally robust to data variability). In order to decide upon the optimal value to take for *M*, we performed random subsample cross-validation and took *M* to be the value that provided the lowest mean misclassification rate across all random subsamples.

### S4. Complete significance tables

Tables 1 and 2 in the main paper were truncated for brevity. In Additional file 1, Tables 1 and 2, we provide complete versions of these tables for the verification and MS data sets respectively.

| Verification data set | | | | | | | |
| --- | --- | --- | --- | --- | --- | --- | --- |
| HAM vs AC | | AC vs U | | HAM vs U | | (HAM & AC) vs U | |
| MW (kDa) | *q*-value | MW (kDa) | *q*-value | MW (kDa) | *q*-value | MW (kDa) | *q*-value |
| 14.7 | 2.1E-02 | 13.8 | 6.2E-04 | 13.8 | 6.7E-04 | 13.8 | 3.4E-05 |
| 13.3 | 2.1E-02 | 6.9 | 5.6E-03 | 11.7 | 6.7E-04 | 6.9 | 1.3E-03 |
| 11.7 | 2.1E-02 | 9.7 | 1.5E-02 | 14.7 | 1.6E-03 | 14.0 | 7.2E-03 |
| 11.9 | 2.1E-02 | 14.0 | 3.6E-02 | 17.4 | 4.0E-03 | 11.7 | 9.9E-03 |
| 8.8 | 2.1E-02 | 8.6 | 3.6E-02 | 79.1 | 4.0E-03 | 8.6 | 9.9E-03 |
|  |  |  |  | 17.6 | 4.0E-03 | 13.9 | 1.3E-02 |
|  |  |  |  | 6.9 | 4.0E-03 | 17.4 | 1.3E-02 |
|  |  |  |  | 39.7 | 4.0E-03 | 17.6 | 1.3E-02 |
|  |  |  |  | 13.3 | 5.3E-03 | 12.8 | 1.7E-02 |
|  |  |  |  | 14.0 | 7.0E-03 | 79.1 | 1.7E-02 |
|  |  |  |  | 8.7 | 1.0E-02 | 39.7 | 1.7E-02 |
|  |  |  |  | 14.1 | 1.2E-02 | 17.3 | 1.7E-02 |
|  |  |  |  | 11.9 | 1.5E-02 | 90.6 | 1.7E-02 |
|  |  |  |  | 17.3 | 1.5E-02 | 9.7 | 1.7E-02 |
|  |  |  |  | 13.9 | 1.7E-02 | 14.7 | 1.8E-02 |
|  |  |  |  | 14.0 | 1.7E-02 | 8.7 | 2.0E-02 |
|  |  |  |  | 47.3 | 1.7E-02 | 14.1 | 2.1E-02 |
|  |  |  |  | 90.7 | 1.7E-02 | 89.0 | 2.5E-02 |
|  |  |  |  | 8.6 | 1.7E-02 | 9.4 | 2.5E-02 |
|  |  |  |  | 12.8 | 2.0E-02 | 14.0 | 3.1E-02 |
|  |  |  |  | 28.2 | 2.1E-02 | 28.2 | 3.4E-02 |
|  |  |  |  | 28.1 | 2.6E-02 | 28.9 | 3.4E-02 |
|  |  |  |  | 56.1 | 2.6E-02 | 15.3 | 3.6E-02 |
|  |  |  |  | 28.9 | 3.0E-02 | 56.1 | 3.6E-02 |
|  |  |  |  | 89.0 | 3.5E-02 | 25.2 | 3.8E-02 |
|  |  |  |  | 25.2 | 4.1E-02 | 13.3 | 3.8E-02 |
|  |  |  |  | 59.0 | 4.3E-02 | 15.1 | 3.8E-02 |
|  |  |  |  | 25.4 | 4.9E-02 | 25.4 | 3.8E-02 |
|  |  |  |  |  |  | 28.1 | 3.9E-02 |
|  |  |  |  |  |  | 47.3 | 3.9E-02 |
|  |  |  |  |  |  | 7.9 | 4.8E-02 |

**Additional file 1, Table S1:** Peaks determined to be significant in the verification data set after correction for multiple testing, (cf. Table 1 in main paper).

| HAM vs MS | |
| --- | --- |
| MW (kDa) | q-value |
| 10.1 | 2.3E-04 |
| 3.8 | 2.3E-04 |
| 7.7 | 2.3E-04 |
| 9.2 | 2.3E-04 |
| 10.3 | 3.3E-04 |
| 37.4 | 3.3E-04 |
| 9.4 | 4.6E-04 |
| 10.8 | 4.8E-03 |
| 14.0 | 4.8E-03 |
| 60.4 | 4.8E-03 |
| 7.5 | 4.8E-03 |
| 28.1 | 4.9E-03 |
| 33.3 | 4.9E-03 |
| 14.7 | 5.5E-03 |
| 6.6 | 7.4E-03 |
| 45.7 | 1.2E-02 |
| 28.3 | 1.3E-02 |
| 91.2 | 1.6E-02 |
| 7.9 | 1.6E-02 |
| 8.5 | 1.8E-02 |
| 13.3 | 2.5E-02 |
| 44.6 | 2.5E-02 |
| 15.8 | 2.8E-02 |
| 66.5 | 3.1E-02 |
| 15.3 | 3.6E-02 |
| 51.2 | 3.6E-02 |
| 79.3 | 3.6E-02 |
| 39.8 | 3.9E-02 |
| 15.1 | 4.3E-02 |
| 16.1 | 4.3E-02 |
| 16.7 | 4.3E-02 |
| 28.9 | 4.3E-02 |
| 6.4 | 4.3E-02 |

**Additional file 1, Table S2:** Peaks determined to be significant in the MS data set after correction for multiple testing, (cf. Table 2 in main paper).

**S5. A simple classification rule using the SELDI data**

We constructed a final classifier to discriminate between HAM and AC using only the 11.7kDa and 13.3kDa peaks, since these peaks were selected consistently in all analyses. For simplicity, rather than using the continuous intensity values, we categorized the observed intensity values for each of these two peaks as either ‘normal’ (encoded by a 0) or ‘abnormal’ (encoded by a 1). ‘Normal’ intensity measurements were defined as those within 2 standard deviations of the mean amongst the uninfected controls; ‘abnormal’ values lay outside these limits. For the 11.7kDa peak, the mean log (intensity) was 3.17 ± SD 0.57; for the 13.3kDa peak the mean was 1.21 ± SD 0.59. We fitted a logistic regression model (by unpenalized maximum likelihood) to the resulting categorical data in order to produce an equation (1) for the probability that a subject randomly drawn from the cohort has HAM:

Pr(Patient has HAM) = 1/(1+exp(-z)) (1)

where z = -1.07 + 3.01 X_11.7_ + 17.58 X_13.3_

Here, X_11.7_ is an indicator variable that is set to zero if the intensity of the 11.7kDa peak is normal, and set to 1 otherwise; X_13.3_ is similarly defined. Note that, according to our fitted model, if X_11.7_ = 0 and X_13.3_ = 1, then Pr(Patient has HAM) = 1.00 (2 decimal places); if X_11.7_ = 1 and X_13.3_ = 0, then Pr(Patient has HAM) = 0.88; if X_11.7_ = 1 and X_13.3_ = 1, then Pr(Patient has HAM) = 1.00; and if X_11.7_ = 0 and X_13.3_ = 0, then Pr(Patient has HAM) = 0.26. If a subject is classified as having HAM whenever Equation (1) yields a result greater than 0.5, then it follows that our model is equivalent to the following simple rule:

“*If the intensity of either the 11.7kDa or the 13.3kDa peak (or both) is abnormal, then classify as HAM”.*

This rule classified 55/68 = 81% of seropositive subjects correctly in the ‘combined’ data set, compared with the random expectation of 50%. This performance level may be overestimated, since the same data were also used to devise the rule. Of the 13 individuals misclassified by the rule, 2 were ACs misclassified as HAM (i.e. false positive diagnosis of HAM), corresponding to a false positive rate of 5.9% and a false negative rate of 32.4%.

### S6. Differences in detection

Our data preprocessing ensured that when we compared two disease classes, and , each peak was detected in at least 40% of the samples from *or* at least 40% of the samples from . This allowed for the possibility that, for example, a peak could be detected in (nearly) all of the samples but (close to) none of the samples. In order to quantify this, we use Fisher’s exact test in order to test for a significant association between detection/non-detection versus membership. We controlled the FDR as before, using the Benjamini-Hochberg procedure. For the original and verification sets we considered each of the comparisons listed in Table 1 (see main paper), and found no significant association between detection/non-detection versus disease class membership. Similarly, with the combined data set there was no significant assocation between detection/non-detection and HAM/AC status. However, for 5 peaks in the HAM/MS data set there was a significant association between disease status (HAM or MS) and detection of the peaks.

**S7. Protein identification: materials & methods**

**Biomarker Identification**

SELDI-TOF-MS was used to guide the purification steps of the selected proteins. To identify these proteins, patient samples containing high quantities of the desired protein were selected. HAM samples were used to identify 11.7, 13.3 and 14.7kDa proteins, whereas AC serum was used to identify the 17.4kDa protein. The number of purification steps was kept to a minimum to avoid any inevitable associated protein loss. Albumin was initially removed from the serum (Albumin Removal Kit, Calbiochem) and the serum then enriched using anionic exchange fractionation column chromatography (Q Ceramic HyperD F beads, BioSepra, US). Fractions were analysed using NP20 chips to monitor the abundance of protein; specifically the biomarkers in question. Fractions containing the desired protein(s) were fractionated again using hydrophobic reverse phase beads (Hydrophobic RPC Poly-Bio beads, BioSepra, US) to remove salts and detergents, before being concentrated and prepared for 1D-SDS-PAGE. Subsequently proteins were either digested in the gel or passively eluted from gels, concentrated, monitored using NP20 arrays, before trypsin digestion. Digested proteins were then analysed using a Q-TOF-MS. All samples were run in duplicate.

**Anionic Exchange Fractionation**

Preferably 100μl serum was depleted of albumin (Albumin Removal Kit, Calbiochem) and concentrated using a speed vac in the presence of a protease inhibitor cocktail (“Complete EDTA-free”, Roche, Germany), before being re-suspended in 100μl of ddH_2_O. Each 100μl reconstituted serum sample was mixed with 150μl of U9 buffer (9M urea, 50mM Tris, 2% v/v CHAPS, pH 9) at room temperature for 30min. Separate mini-columns (Bi Mo BiTec, Goettingen, Germany) for each sample were prepared during the incubation, by loading a final volume of 200μl of Q Ceramic HyperD F beads (BioSepra, US) into a 1ml mini-spin column. Excess fluid from the beads was removed using a disposable 5ml plastic syringe. The beads were subsequently equilibrated by the addition of 500μl of U1 buffer buffer (1M urea, 50mM Tris, 2% v/v CHAPS, pH 9) and vortexing for 10min. The buffer was then discarded using a plastic syringe and another 500μl of U1 buffer added and mixed for an additional 10min. After the 30min incubation of the sample and U9 buffer, 250μl of U1 buffer was added to each sample and briefly vortexed. Each sample (500μl in total) was then applied to its specific column and mixed gently for approximately 30min.

Using a plastic syringe, the flow through was collected. The column was then washed with 500μl of 50mM Tris-HCl, pH 9.0, and mixed gently again for 10min. The first fraction (referred as fraction 1) was collected in the same manner as described for the flow through. The column was washed with 500μl of 50mM HEPES, pH 7.0, gently mixed for 10min and the second fraction (fraction 2) was collected. This process was repeated using 100mM sodium acetate buffers at pH 5.0 and 4.0, 20mM sodium citrate, pH 3.0 and an acid/organic buffer (33% Isopropanol, 16.7 % ACN, 0.1% TFA), so that in total (including the flow through) seven fractions were collected from the original sample. 1μl of each of the collected fractions was spotted onto NP20 chips, allowed to air dry, before being analysed on the SELDI machine using SPA.

# Reverse Phase Fractionation

Hydrophobic RPC Poly-Bio beads (BioSepra, US) were added to a mini-spin column (Mo BiTec, Goettingen, Germany) and equilibrated with 50μl of 10%ACN/0.1% TFA. Samples were adjusted to contain a final concentration of 10%ACN/0.5% TFA, before their addition to the mini-spin column containing the beads. Samples were incubated with the beads, vortexed for 30min at room temperature, and subsequently centrifuged for 1min at 5000rpm and flow through collected. This fraction should contain the salts and detergents. Proteins were then eluted by the addition of 400μl of 10%, 20%, 30%, 40%, 50%, 70% and 90% ACN/0.1% TFA. Eluted proteins were profiled on NP20 arrays using SPA. If protein fractions were to be desalted after anionic fraction, all proteins were generally eluted using a single addition of 90% ACN/0.1% TFA before concentration by speed vac and purification via SDS PAGE.

**1D SDS PAGE**

SDS PAGE was carried out using the NuPage XCell SureLock Mini-Cell system from Invitrogen, US. Fractionated and concentrated serum samples were reconstituted in 2μl of concentrated ammonium hydroxide (no more, otherwise wells in the gel may collapse) followed by 7.5μl NuPage LDS sample buffer (4X) and 20.5μl ddH_2_O for Bis-Tris gels, or 15μl Novex Tricine sample buffer (2X) and 13μl ddH_2_O for Tricine gels. Samples were gently mixed by vortexing. The pH of samples was corrected to 7 accordingly. Samples were allowed to stand at room temperature for 1 hr before being heated and loaded directly onto the gel. SDS PAGE was subsequently carried out typically using 12% Bis-Tris gels or 16% Tricine gels, according to the manufacturer’s recommendations. Gels were run with the Mark 12^TM^ Unstained molecular weight marker (Invitrogen, US, markers from 200 – 2.5kDa).

**Gel Staining**

All gels were stained using Colloidal Coomassie (Invitrogen Simply Blue). Gels were washed 3 times in ddH_2_O for 5 min per wash before being placed in approximately 20ml of Colloidal Coomassie stain and incubated for 1 hr on a rocking table at room temperature. Subsequently gels were rinsed with several washes of ddH_2_O and destained for 1 hr in ddH_2_O.

**Gel Band Excision**

Gels were placed on a glass or plastic cutting board that had been freshly cleaned using methanol. The target molecular weight of proteins to be identified (revealed by the SELDI profiling) was located on the gels. Visible bands at the target molecular weight were carefully excised using a new and clean scalpel blade. In addition (and if bands were not clearly visible) small thin gel slices above and below the desired molecular weight were excised. All gel slices excised were kept to the same size and cut into small cubes (approximately 1mm^3^ gel pieces). The gel pieces were carefully placed into methanol washed 500μl Eppendorf tubes. The remaining gels were kept in case of further gel band excision by placing them in sealed plastic containers in ddH_2_O at 4°C for up to 2 weeks.

**In-Gel Protein and Passive Elution Digestion**

The excised gel pieces were initially washed on a shaker for at least 30min in a solution of 40% Methanol/10% acetic acid, until the Coomassie stain and SDS had been removed. Gel pieces were then washed twice in 200μl 30% acetonitrile/100mM ammonium bicarbonate (pH 8, prepared fresh) solution for 30min and subsequently dehydrated with 50μl 100% acetonitrile for 15min and dried at 50°C.

Proteins were then either digested in-gel or passively eluted. For in-gel digestion, dried gel pieces were rehydrated with 10-20μl of 20mM ammonium bicarbonate containing 20ng/μl of mass spectrometry grade trypsin (Roche, Germany). The gel pieces were allowed to swell and an additional 10-30μl of 20mM of ammonium bicarbonate (pH 8) was added so that the gel pieces remained covered by solution (the smallest volume possible). Once rehydrated, the gel pieces were incubated for 16 hours at 37°C before Q-TOF analysis.

The passive elution was performed by the addition of 10 - 20μl (just enough to cover the gel pieces) of 45% formic acid/30% acetonitrile/10% isopropanol to the dry gel pieces, and proteins were eluted into solution. Samples were then shaken for 30min before being incubated for 4 hours at room temperature (if it was necessary additional 45% formic acid/30% acetonitrile/10% isopropanol was added to the samples). At this stage 1μl of the passive elutant from each sample was spotted on to an NP20 chip, heated at 60°C for 5min and analysed using the SPA matrix by SELDI-TOF-MS to ensured that the correct protein had been purified. The remaining passive elution was incubated over-night. The solution was transferred to a fresh Eppendorf tube and the gel pieces were discarded. The passive elution solution was pelleted in a Speedivac, and the supernatant discarded. The pellet was reconstituted in 5μl of 20ng/µl Trypsin (Promega) in 25mM ammonium bicarbonate/10% acetonitrile and subsequently incubated at 37°C for 4 hours. Samples were analysed using Q-TOF-MS (performed by Nathan Harris at Ciphergen (Guildford, Surrey, UK) and/or Robin Wait at the Kennedy Institute of Rheumatology (Imperial College, London, UK)).

**S8. Prediction using ‘original’ as training, ‘verification’ as test**

As an alternative method for assessing the validity of our results, we additionally considered using the ‘original’ dataset as a training set for a predictive multivariate model, which we then tested on the ‘verification’ set. Although this has the effect of halving the amount of data available to fit the model, it also allows us to avoid using the data more than once. We applied the method of Additional file 1, S3 to the ‘original’ dataset . For *M* = 1, the 11.7kDa peak was selected (with mean CV misclassification rate 14.1%); for *M* = 2, the 11.7kDa and 13.3kDa peaks were selected (with mean CV misclassification rate 14.6%); for *M* = 3, the 11.7kDa, 13.3kDa and 17.4kDa peaks were selected (with mean CV misclassification rate 18.9%); and for *M* > 3 the mean CV misclassification rate was >20%. Following our method for choosing *M*, the 11.7kDa peak was taken as our final selection. However, we also considered the 11.7kDa and 13.3kDa peaks together, since the mean CV misclassification rates for *M* = 1 and *M* = 2 were very similar. We therefore trained two models on the ‘original’ dataset: (1) an unpenalised logistic regression model using just the 11.7kDa peak as the only predictor; and (2) an unpenalised logistic regression model using both the 11.7kDa and 13.3kDa peaks as predictors. We applied the 2 trained models to the ‘verification’ dataset, in both cases resulting in a correct classification rate of 70.6%. We additionally plotted ROC (receiver operating characteristic) curves for both models, as shown in Additional file 1, Figure 1. From this, we calculated the AUC (‘area under curve’), yielding 0.80 for Model (1) and 0.83 for Model (2). Given that the ‘original’and ‘verification’ datasets were collected at different times, and processed separately from one another, this good performance lends further support to the robustness and validity of the 11.7kDa and 13.3kDa peaks as predictors of HAM/TSP.


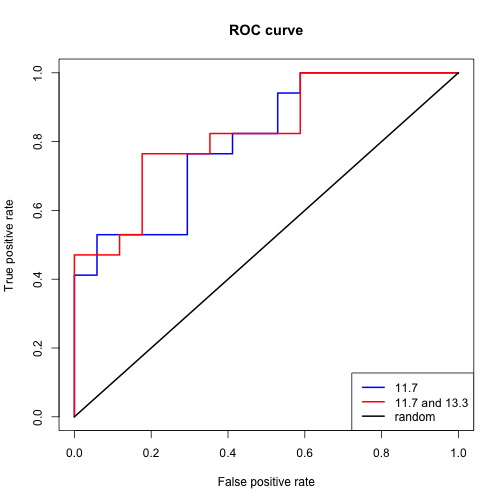


Additional file 1, Figure 1: ROC curves for two unpenalized logistic regression models trained on the ‘original’ dataset, with performance evaluated on the ‘verification’ dataset. The blue ROC curve is for the model that uses only the 11.7kDa peak as a predictor, while the red curve is for the model that uses both the 11.7kDa and 13.3kDa peaks as predictors. The black line indicates the expected performance under a random classifier.

**S9. Workflow summary**


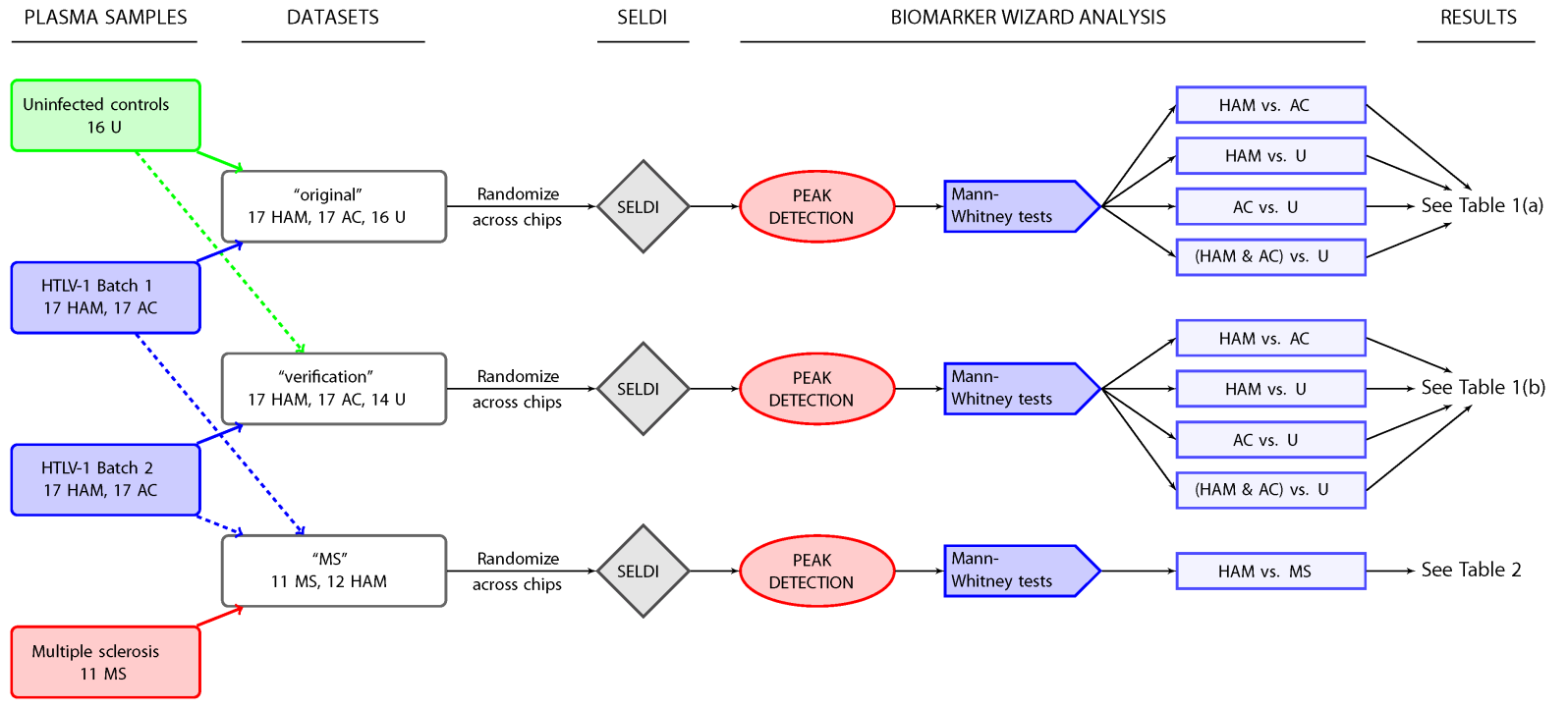


**Figure S9a:** Experimental design and data processing workflow in the univariate analysis. Collected plasma samples were analysed in 3 datasets (“original”, “verification” and “MS”). Dashed lines indicate that only a subset of the collected plasma samples were included in the corresponding dataset (see text for details). After randomization across chips, samples were analysed by SELDI mass spectrometry. Peak detection and univariate analysis were performed using Biomarker Wizard and the results (after correction for multiple comparisons) are summarized in Tables 1 and 2.


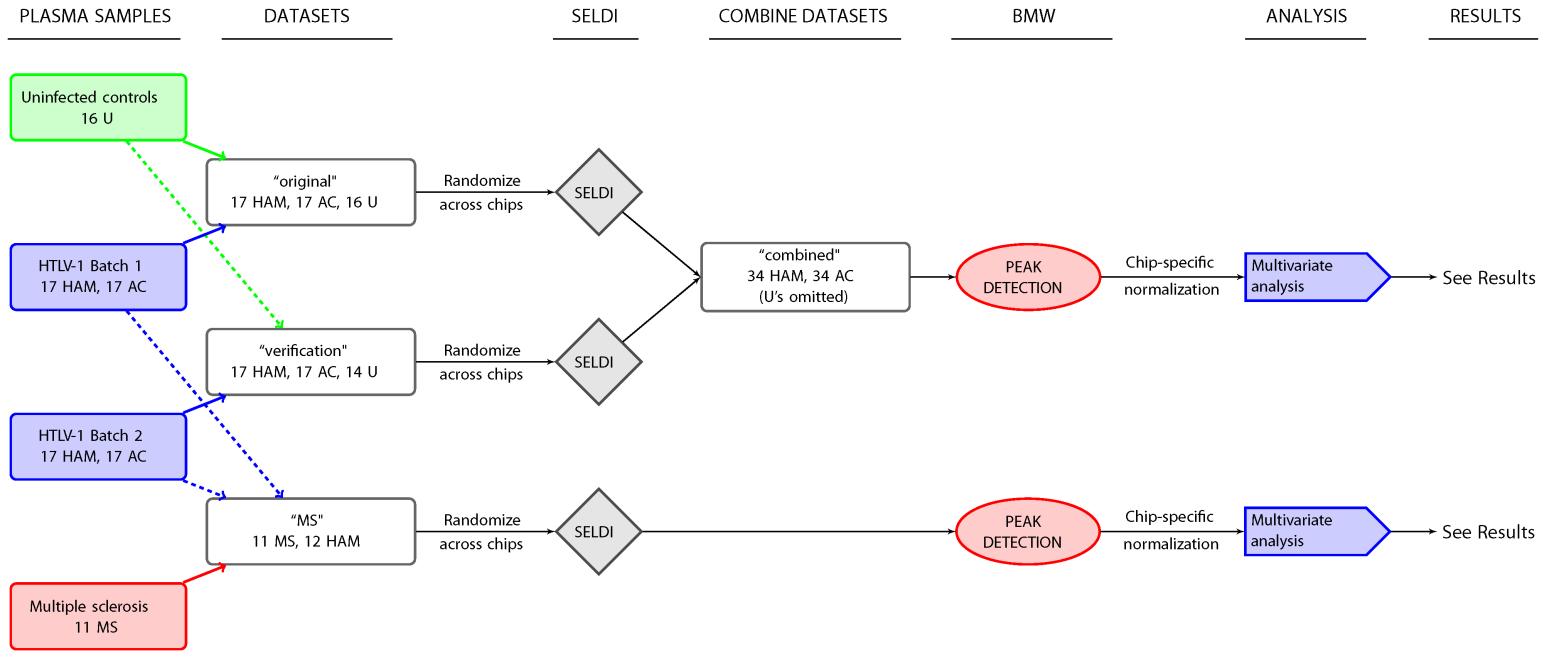


**Figure S9b:** Experimental design and data processing workflow in the multivariate analysis. Here, the HTLV-1 samples from the “original” and “verification” sets were combined before peak detection (using Biomarker Wizard (BMW)) and statistical analysis.

**References**

1. Issaq HJ, Veenstra TD, Conrads TP, Felschow D: **The SELDI-TOF MS approach to proteomics: protein profiling and biomarker identification.** *Biochem Biophys Res Commun* 2002, **292:**587-592.

2. Agranoff D, Fernandez-Reyes D, Papadopoulos MC, Rojas SA, Herbster M, Loosemore A, Tarelli E, Sheldon J, Schwenk A, Pollok R, et al: **Identification of diagnostic markers for tuberculosis by proteomic fingerprinting of serum.** *Lancet* 2006, **368:**1012-1021.

3. Petricoin EF, Ardekani AM, Hitt BA, Levine PJ, Fusaro VA, Steinberg SM, Mills GB, Simone C, Fishman DA, Kohn EC, Liotta LA: **Use of proteomic patterns in serum to identify ovarian cancer.** *Lancet* 2002, **359:**572-577.

4. Ndao M, Rainczuk A, Rioux M-C, Spithill TW, Ward BJ: **Is SELDI-TOF a valid tool for diagnostic biomarkers?** *Trends in Parasitology* 2010, **26:**561-567.

5. Baggerly KA, Morris JS, Coombes KR: **Reproducibility of SELDI-TOF protein patterns in serum: comparing datasets from different experiments.** *Bioinformatics* 2004, **20:**777-785.

6. Churchill GA: **Using ANOVA to analyze microarray data.** *Biotechniques* 2004, **37:**173-175, 177.

7. Tibshirani R: **Regression shrinkage and selection via the Lasso.** *Journal of the Royal Statistical Society Series B-Methodological* 1996, **58:**267-288.

8. Friedman J, Hastie T, Tibshirani R: **Regularization paths for generalized linear models via coordinate descent.** *J Stat Softw* 2010, **33:**1-22.

9. Meinshausen N, Buehlmann P: **Stability selection.** *J Roy Stat Soc B* 2010, **72:**417-473.
